# Examining the challenges of people living alone with neurodegenerative conditions: a scoping review protocol

**DOI:** 10.1136/bmjopen-2024-090146

**Published:** 2025-06-10

**Authors:** Anthony Martyr, Maria Caulfield, Catherine Charlwood, Laura D Gamble, Matthew Prina, Jan R Oyebode, Claire Hulme, Linda Clare

**Affiliations:** 1 Department of Health and Community Sciences, University of Exeter Medical School, Exeter, UK; 2 NIHR Policy Research Unit in Dementia and Neurodegeneration University of Exeter (DeNPRU Exeter), Exeter, UK; 3 Centre for Applied Dementia Studies, University of Bradford, Bradford, UK; 4 Wolfson Centre for Applied Health Research, Bradford, UK; 5 NIHR Applied Research Collaboration South-West Peninsula, Exeter, UK; 6 Population Health Sciences Institute, Newcastle University, Newcastle upon Tyne, UK

**Keywords:** Dementia, Motor neurone disease, Dementia, Parkinson-s disease, Review

## Abstract

**Abstract:**

**Introduction:**

People living alone with neurodegenerative conditions face unique difficulties in maintaining independence and accessing appropriate health and social care support. Consolidating current understanding regarding these unique difficulties would better inform health and social care services and enable more tailored and appropriate service delivery. The proposed scoping review will summarise evidence from studies that provide evidence about people with dementia, Parkinson’s disease, Huntington’s disease or motor neurone disease who live alone. This protocol sets out the processes that will be followed in the subsequent scoping review to ensure that a transparent, rigorous and reproducible approach is used to identify, select and synthesise relevant evidence.

**Methods and analysis:**

This scoping review protocol uses well-established methodology outlined by the Preferred Reporting Items for Systematic review and Meta-Analysis and the Joanna Briggs Institute. Relevant publications will be searched using PubMed, Web of Science Core Collection, CINAHL and AgeLine via EBSCOhost and EMBASE, PsycInfo and Social Policy and Practice via Ovid. Grey literature will be searched via Google looking specifically for pdf documents. As there was no previous review on the topic, no date restrictions will be applied to the searches. Piloting of the search strategy provided an estimate of the number of titles likely to require title and abstract screening, which, along with prior experience from a similar review approach, informed the feasibility of the proposed strategy. For research publications, a two-stage screening approach will be undertaken. The first stage will involve screening titles and abstracts for relevant literature on people with neurodegenerative conditions living alone in the community. The second stage will involve full text screening of selected articles. For grey literature, the first 20 PDFs per website identified in Google will be downloaded and screened. Summary data will be extracted from publications selected for inclusion. Data synthesis will involve tabulating study characteristics and findings and preparing narrative summaries to identify commonalities, gaps and areas for future research.

**Ethics and dissemination:**

Ethical approval is not required for this review, as the information included is in the public domain and people with lived experience are consultees rather than research participants. Consultation with people with lived experience, stakeholders and experts linked with the National Institute for Health and Care Research Policy Research Unit in Dementia and Neurodegeneration University of Exeter will help to ensure the relevance and applicability of findings. Dissemination will include a policy report and peer-reviewed publications aimed at informing policy, practice and improving support services for people living alone with neurodegenerative conditions.

**STRENGTHS AND LIMITATIONS OF THIS STUDY**

- The use of a rigorous and well-established methodological framework will enhance the quality and structure of the review.
- A comprehensive search in seven databases will maximise identification of all possible records that meet the inclusion criteria of the review.
- The inclusion of grey literature will complement the research literature by providing detail about guidance being given to people living with and people caring for neurodegenerative conditions.
- A limitation is that the literature search will be restricted to articles published in English. This increases the risk that relevant information will be missed.

## Background

 Since the 1960s, the proportion of adults living alone has steadily increased.^1^ In the UK, census data show that, in 2021, 30.1% of adults lived alone,^2^ and this proportion is largely consistent with other wealthy nations in Europe and North America.^1^ Living alone has become more common due to societal changes and shifts in lifestyle preferences. Factors such as increased urbanisation, delayed marriage, higher divorce rates and a focus on individualism and personal autonomy have all contributed to the rise in the number of adults living alone.^3^ These factors largely affect younger or middle-aged adults, whereas different factors are likely to be more salient for older adults. In later life, living alone is often the result of life transitions such as the death of a spouse or partner and the departure of children from the household. Women are particularly likely to live alone in old age,^4^ as they tend to live longer than men and are more often widowed.^5^ As life expectancy increases, many older people are spending longer periods living alone in later life. This is important given that living alone is increasing most rapidly among older people.^6 7^

As the proportion of people living alone continues to increase, it is to be expected that there will be a concomitant increase in the number of people living alone in the community with a neurodegenerative condition. The four most common neurodegenerative conditions are Alzheimer’s disease and other types of dementia, Parkinson’s disease, Huntington’s disease and motor neurone disease,^8^ including amyotrophic lateral sclerosis or Lou Gehrig’s disease. Rarer neurodegenerative conditions include prion diseases like Creutzfeldt-Jakob disease, multiple system atrophy, progressive supranuclear palsy, corticobasal syndrome and ataxia. Most of these conditions are associated with increased age and are more prevalent in people over 65 years of age, though Huntington’s disease, motor neurone disease and some types of dementia often appear in people aged under 65 years. It is estimated that worldwide there are over 55 million people living with dementia,^9^ more than 10.9 million people living with Parkinson’s disease,^10^ around 400 000 people living with Huntington’s disease^11^ and around 260 000 people living with motor neurone disease.^10^ Precise population-based estimates of the number of people living alone in the community with these conditions in the UK are lacking. Gould *et al*^12^ estimated that, in the USA, in 2011, 13% of people with dementia were living alone in the community. Where research studies have recruited samples that include people with dementia living alone or with others in the community, the proportion of people living alone varies considerably, from 18.5% in a cohort study recruited in Britain^13 14^ to 51% in a German sample.^15^ These differences are likely to be due to variation in sampling methods. One online study that included nearly 5500 people with Parkinson’s disease and recruited globally, though around 80% were from the USA, reported that 12% lived alone.^16^

People living alone with neurodegenerative conditions may require more support to maintain independence and to continue to live a safe and fulfilling lifestyle than those living with others. Most people living alone with neurodegenerative conditions have access to varying degrees of help and support from unpaid or informal carers, typically family or friends.^12 13 17^ Indeed, it is widely assumed that a family carer is available to provide support to the person if needed and act as a source of information for health and social care professionals. Where a family carer is not available to assist, some studies suggest people with dementia may have difficulties in engaging and interacting with services and may find services unresponsive,^18^ making it more challenging for people to manage their condition independently. Additionally, people with Parkinson’s disease find services difficult to access.^19^ These challenges may increase as the severity of the condition progresses. Over time, the service use of people living with these conditions tends to rise,^20^ with costs for people with dementia living alone being around 35% higher over 2 years compared with those living with others.^21^ As the number of people living alone with neurodegenerative conditions increases, this will inevitably escalate the demands placed on healthcare service providers and stretch service capacity.

Evidence suggests that people who live alone with neurodegenerative conditions are at an increased risk of numerous adverse outcomes. These include increased social isolation, increased loneliness, lower satisfaction with life, greater financial exploitation such as falling victim to scams or being coerced into altering legal or financial documents, higher incidence of accidental injury, elevated risk of malnutrition, increased psychological distress, greater self-neglect and a higher rate of admission to residential care.^12 14 19 22^ Therefore, as the number of people living alone with neurodegenerative conditions continues to rise, it is important to identify the unique challenges to living alone with these conditions, as this will enable people to live as independently as possible for as long as possible. Furthermore, it is essential to develop comprehensive strategies within healthcare systems to meet their needs, as addressing these needs not only enhances quality of life but also reduces the burden on healthcare systems through, for example, preventing crises and avoidable hospital admissions. This, in turn, promotes more efficient resource allocation and improved outcomes.

### Study rationale

It is important to consider how people with neurodegenerative conditions cope when living alone, especially as the number of people living alone with a neurodegenerative condition is increasing and family carers are not always available. Understanding the diverse needs and challenges associated with living alone with each condition can inform policy and, in turn, influence how services are structured, planned and delivered. This applies to both the voluntary sector and health and social care provision for people living with a neurodegenerative condition, as well as practitioner training and skills development.

While most research on this topic focuses on dementia, by considering this issue across a range of neurodegenerative conditions, we aim to highlight differences and similarities in care delivery and needs. This can help to identify good practice and generate recommendations for improvement in care and support services for people living alone with these conditions and identify what barriers they encounter to maintain their independence. To do this, we need to understand the needs and challenges that people experience when living alone, gain an overview of how services are provided, identify gaps in care coordination and integration and provide recommendations for training and education in the health and social care workforce. Synthesising available evidence is expected to result in recommendations that can inform the development of policies and services tailored to meet the diverse needs of people with neurodegenerative conditions who live alone.

To date, there have been no comprehensive systematic reviews or scoping reviews analysing the extant literature on people who live alone with neurodegenerative conditions. Using a scoping method allows for a thorough and systematic review process while also providing flexibility to incorporate various types of studies, such as quantitative, qualitative, mixed methods and case studies. Conducting a scoping review on this topic will provide insights into the challenges, coping mechanisms and support systems of people living alone with neurodegenerative conditions. The review will help to identify gaps in our understanding and where future research should be directed. It will enable us to inform policy and practice and identify key areas of health and social care where changes would provide the most benefit.

### Study Objectives

The aim of this scoping review is to systematically assess both research and grey literature concerning people with neurodegenerative conditions who live alone in the community. The primary aim of the review will be to identify the characteristics of people living alone, their experiences, needs, challenges, resource use and associated costs to the health and social care sectors, individuals and families and how these change over time. We will also evaluate current practice from practice guidelines, information for practitioners and practical advice for people with different neurodegenerative conditions. This will show what information is available, what practitioners are doing, and what might constitute good or best practice. In addition, we will consider inequities experienced by people with neurodegenerative conditions who live alone relative to those living with others, which may include issues related to social isolation and exclusion, access to health and social care services and support from carers. We will use the evidence synthesised in the review to generate recommendations for policy and practice and highlight any gaps in knowledge that may represent areas for future investigation.

## Methods and analysis

The approach used in this document follows the protocol methodology outlined in Preferred Reporting Items for Systematic review and Meta-Analysis Protocols (PRISMA-P) guidance,^23 24^ and Joanna Briggs Institute best practice guidance for scoping reviews.^25^ A completed PRISMA-P checklist can be found in online supplemental table 1.

### Eligibility criteria

The following types of sources will be included:

- Studies that report information about people with neurodegenerative conditions who live alone; this includes both studies that focus solely on people living alone and studies that compare people living alone with people living with others.
- Studies that focus on people with dementia, Parkinson’s disease, Huntington’s disease or motor neurone disease. Studies reporting on rarer neurodegenerative conditions will also be included. It is anticipated that most studies will involve people with dementia, with fewer studies covering other neurodegenerative conditions.
- Studies where samples are described as having ‘cognitive impairment’ are likely to include people who have dementia but lack a formal diagnosis. These will typically involve older people with undiagnosed dementia or significant cognitive impairment who rarely contact health services. Such individuals can only be reported descriptively as having ‘cognitive impairment’ and those without a definite diagnosis may be in the greatest need of support, making policy direction most urgently required.
- Studies that report on mixed diagnostic groups where cognitive impairment is a factor (eg, studies that include those with neurodegenerative conditions but also people with stroke, head injury or other acute insults). These studies will be included if one of the following conditions is met:
  1. They provide information about people with relevant conditions separately.
  2. If conditions are not reported separately, at least 80% of the sample must include people with relevant neurodegenerative conditions, provided other inclusion criteria are met.
- Grey literature that offers support or advice to people living alone with neurodegenerative conditions, their carers or health and social care professionals.
- Grey literature that addresses topics of relevance to people living alone with neurodegenerative conditions, such as managing autonomy and risk, loneliness, ensuring home safety, planning for future care, etc.

The following types of sources will be excluded:

- Studies focusing on people diagnosed with mild cognitive impairment. These people do not have dementia, and most people diagnosed with mild cognitive impairment either remain stable or revert to normal cognitive functioning for their age.^26^ This group differs from the above ‘cognitive impairment’ group as mild cognitive impairment is a specific diagnosis.
- Studies focusing on people diagnosed with multiple sclerosis, as this is considered an autoimmune disease rather than a neurodegenerative condition.
- Studies focusing on people classified as having ‘cognitive impairment not dementia’ or ‘CIND’, as while these people have cognitive impairment, dementia has specifically been excluded.
- Studies that report on people with cognitive impairment due to head injury, stroke or other acute insults.
- Grey literature that refers to living alone within the document but lacks substantial commentary, advice or relevance to the experience or needs of people living alone with a neurodegenerative condition and/or their carers.

Other exclusion criteria are:

- Articles written in languages other than English.
- Articles for which full text versions are unavailable.
- Reviews, editorials, letters and opinion pieces.
- Published conference abstracts; these will be used to find subsequently published articles where available. When articles are found via this route, they will be included in the ‘identified via other sources’ section of the PRISMA flowchart.
- Studies describing mixed samples that do not meet the inclusion criteria will be excluded. This includes studies where:
  - The sample includes people with one or more of the included conditions (eg, dementia) along with one or more conditions from the excluded groups (eg, mild cognitive impairment), but less than 20% of the sample comprises people with relevant conditions, and data are not reported separately for different conditions.

### Information sources

Relevant studies will be identified by searching seven electronic databases covering published research literature: PubMed, Web of Science Core Collection, CINAHL and AgeLine via EBSCOhost and EMBASE, PsycInfo and Social Policy and Practice via Ovid. The choice of these information sources was informed by prior discussions with expert librarians, the experience of the review team and methods employed in a previous review undertaken by the team.^27^ As there have been no comprehensive systematic reviews or scoping reviews analysing the extant literature on people who live alone with neurodegenerative conditions, no date restrictions will be applied to the searches.

Grey literature including reports, information booklets, advice leaflets, practice guidelines, etc, will be searched using advanced Google search parameters that will be applied to 76 preselected websites; a list of these 76 websites can be found in online supplemental table 2. The 76 websites have been selected as they are either the main English language condition-specific websites for dementia, Parkinson’s disease, Huntington’s disease or motor neurone disease, hosted by organisations in the UK, the USA, Canada, Europe or Australia or websites of widely known global health organisations or health and social care think tanks. Grey literature searches will be restricted to PDF documents only. As the most applicable PDF documents will be returned at the top of the search engine results page, the first 20 PDF documents that Google returns for each website will be downloaded and screened. Where the search returns fewer than 20 PDF documents, all PDF documents that are returned will be screened.

### Search strategy

The search terms to be used were collated by screening recent reviews of other conditions that have used synonyms for living alone. Search terms will be restricted to English language publications when databases permit using this restriction. No other restrictions will be applied.

Search terms comprise two components: a string comprising terms related to living alone and a neurodegenerative condition string; see box 1.

Box 1 **Search string components**

- Living alone string terms

Living alone OR Live* alone OR Single-living OR One-person household OR Singlehood OR Single people OR Single person OR Single men OR Single women OR solo

- Neurodegenerative condition string terms

dement* OR Alzheimer* OR Parkinson* OR Lewy OR Fronto* OR Huntington* OR Chorea OR amyotrophic lateral sclerosis OR ALS OR motor neuron* disease OR MND OR progressive muscular atrophy OR Gehrig OR neurodegen* OR neurolog* OR cognitiv* impair*

- Example search string for Ovid

((dement* or Alzheimer* or Parkinson* or Lewy or Fronto* or Huntington* or Chorea or amyotrophic lateral sclerosis or ALS or motor neuron* disease or MND or progressive muscular atrophy or Gehrig or neurodegen* or neurolog* or cognitiv* impair*) and (Living alone or Live* alone or Single-living or One-person household or Singlehood or Single people or Single person or Single men or Single women or solo)).ti,ab.

See online supplemental table 3 for the search strings used in each database. Piloting of the search strategy was used to determine the feasibility of the search terms and databases, alongside prior experience with a similar review approach.^27^

The search strings will be adapted when searching for the grey literature. Different search terms will be used for condition-specific and non-condition-specific websites. For condition-specific websites, the search will use these living alone synonyms only: “Living alone” OR “Live alone” OR “Lives alone” OR “Lived alone”. An example search string for a condition-specific website is: site:alzheimers.org.uk “Living alone” OR “Live alone” OR “Lives alone” OR “Lived alone” filetype:pdf.

For non-condition-specific websites, searches will be conducted using each of the four search terms separately, for example, “Living alone” OR “Live alone” OR “Lives alone” OR “Lived alone”, combined with an adapted list of the condition terms used in the published literature search described above. An example search string is: site:who.int “living alone” dementia OR demented OR Alzheimer OR Parkinson OR Lewy OR Fronto OR Huntington OR Chorea OR “amyotrophic lateral sclerosis” OR ALS OR “motor neuron disease” OR MND OR “progressive muscular atrophy” OR Gehrig OR neurodegenerative OR neurodegeneration OR “cognitive impairment” OR “cognitively impaired” filetype:pdf.

### Data management

Literature search results will be imported into EndNote. Duplicates will be removed using a series of duplicate searches within EndNote. A final manual screen for any additional duplicates will be conducted. Title, abstract and full text screening stages will be undertaken within EndNote. For grey literature searches, PDF documentpdfs will be downloaded into folders, one folder for each website searched. Details of each grey literature search will be recorded in an Excel spreadsheet including the search date, the name of the researcher who conducted the search, the search string(s) used for each website, the number of PDF documentpdfs returned in the search engine results page, the number of duplicates manually removed and the number of PDF documentpdfs extracted for screening.

### Selection process

The selection processes for research and grey literature are described separately below. Reasons for exclusion at the full text stage will be recorded and reported. Records of all searches will be recorded and summarised using PRISMA guidelines and recommendations for reporting; a PRISMA flowchart summarising the screening stages will be included.

#### Research literature

The study selection process will comprise two levels of screening. Following the removal of duplicates, titles and abstracts of all returned records will be screened by two reviewers working independently. Any records that are deemed potentially relevant by either or both reviewers will be taken forward to the next stage. In this second stage, the full text of each record will be downloaded and examined against eligibility criteria for inclusion in the review. Full texts will be screened for eligibility by one researcher. To check for consistency, 20% of these will be screened by a second researcher, blinded to the decision of the first researcher. Where it is not clear whether articles should be included, decisions about eligibility will be made via group discussion involving a third reviewer. Neither journal titles nor author names will be blinded during any screening stage.

Where randomised controlled trials are included, only information from the baseline analysis will be used, except where the trial reports intervention effects separately for people living alone and people living with others, in which case data from follow-up assessments will also be used.

Multiple articles that use data from the same study will be identified and collated to avoid duplication of findings. Where the same variables are included in multiple articles from the same study, preference will be given to the article that reports the largest sample size. Where sample sizes are identical and where the same variables are included, data from the most recent article to be published from the same study will be used.

#### Grey literature

For grey literature, a similar procedure will be used. As items of grey literature may not contain abstracts, the full text of all PDF documents that are downloaded will be screened following the full text screening procedure outlined above for research articles. Any research articles or published conference abstracts identified in the grey literature search will be cross-checked with the research literature search and either excluded if already found or screened as described above for the research literature. For recording purposes, any articles identified in this way will be included in the ‘identified via other sources’ section of the PRISMA flowchart.

### Data collection process and data items

Data extracted from research and grey literature will be recorded using Excel spreadsheets.

#### Research literature

The study characteristics to be extracted will include several key areas. First, information about the study will be recorded. This information will comprise: first author name, publication year, study methodology, date that study data were collected, study name or funding grant number to allow identification of multiple articles that use data from the same study and country or countries where data collection took place.

Next, details about the sample or population will be documented. This will comprise: participant age, sex, education, ethnicity, diagnosis, diagnostic criteria used, severity—either cognitive screening mean score or other condition-specific severity metric such as Hoehn and Yahr stage and/or Unified Parkinson’s Disease Rating Scale score and/or number of people in severity bands and number and/or proportion of people with a neurodegenerative condition.

Information about the living situation will also be extracted. This will comprise the number and/or proportion of people living alone and other included groups that are not living alone.

In addition, data extraction will record information about variables relevant to the research questions regarding living alone as well as any recommendations for policy or practice proposed by the study authors.

The following procedures will be followed for both quantitative and qualitative findings. For quantitative studies, data will be extracted for all variables relevant to living alone; this will comprise both data relating to findings and summary statistics. Where comparisons are made, relevant data will be extracted for people living alone and for people not living alone.

For qualitative studies, overarching themes and subthemes, representative quotations where applicable, and main findings related to living alone will be extracted.

No quantitative nor qualitative studies will be excluded where some of this information is missing; where this is the case, data that are missing will be clearly identified.

#### Grey literature

For grey literature, the following information will be extracted. Information about the document: publication year, organisation on behalf of which the publication was produced, author, document type, country of origin, specific context surrounding discussions on living alone.

Where relevant, the processes used to gather information, such as surveys, interviews and consultation, etc, will be noted. Any information provided in the document about living alone with a neurodegenerative condition will be recorded, along with any advice, support, guidance or recommendations provided in the document for policymakers, practitioners or people living with the condition.

In addition, any recommendations aimed at improving treatment and care for people living alone with neurodegenerative conditions will be recorded, as well as any other potentially relevant information.

### Data synthesis

Data will be presented in tables that describe the study characteristics and the findings for each included study. There will be separate tables for quantitative and qualitative research literature and for grey literature. Mixed methods studies will be tabulated in applicable tables where relevant or in a separate table. The proportion of people living alone with each condition will be calculated and presented. A narrative summary will accompany the table of results and will describe how the results relate to the review aim and research questions. Findings will be grouped and described in the results section.

The Campbell and Cochrane Economic Methods Group Brief Economic Commentary framework^28^ will be used to summarise the main findings of studies reporting health economic data.

Results will be reported in line with the PRISMA scoping review extension.^29^

### Patient and Public Involvement

Stakeholders and people with lived experience have been involved in developing plans for this review. There are two experts by experience on the project team—a former carer at a distance for a parent living alone with dementia and a person living alone with Parkinson’s disease. They were involved in project team discussions about the nature of the review. Once review findings are available, further consultation will be undertaken to examine these in relation to lived experience and practice and help to ensure resulting recommendations are relevant and realistic. Stakeholder consultation will draw on the expertise within the DeNPRU Exeter Knowledge Exchange Community (KEC), which includes experts by experience alongside other stakeholders. Online workshops will be advertised to our community and by the members of the KEC. Additional specific expertise will be sought where needed. As the contributors to these discussions are consultees who have volunteered to support the project by sharing their opinions and experiences and not recruited as research participants, research ethics committee approval is not required, as University guidance confirms. All experts by experience will be paid in recognition of their time and effort.

### Dissemination and ethics

Three articles will be written up for publication based on the literature review, one for the quantitative literature, one for the qualitative literature and one about the stakeholder consultations. The findings will form part of a wider project on the topic conducted by the National Institute of Health and Care Research Policy Research Unit in Dementia and Neurodegeneration University of Exeter. The findings will also be used in the policy reports and policy briefs arising from the project. Ethical approval is not required for this study. The review procedures described in this protocol are expected to be completed by the end of November 2024.

## Supplementary material

10.1136/bmjopen-2024-090146 online supplemental file 1
