# Supplementary material for: Examining the challenges of people living alone with neurodegenerative conditions: a scoping review protocol
Source: BMJ Open. 2025 Jun 10;15(6):e090146. doi: 10.1136/bmjopen-2024-090146 (PMC12161305; doi:10.1136/bmjopen-2024-090146)
Supplement: online supplemental file 1 [file bmjopen-15-6-s001.docx]

Examining the challenges of people living alone with neurodegenerative conditions: a scoping review protocol. Supplementary Tables

Anthony Martyr^1,2^, Maria Caulfield^2,3^, Catherine Charlwood^1,2,5^, Laura D. Gamble^2,6^, Matthew Prina^2,6^, Jan R. Oyebode^2,3^, Claire Hulme^1,2^, & Linda Clare^1,2,5^

1. Department of Health and Community Sciences, University of Exeter Medical School, Exeter, UK
2. NIHR Policy Research Unit in Dementia and Neurodegeneration University of Exeter (DeNPRU Exeter), UK
3. Centre for Applied Dementia Studies, University of Bradford, Bradford, UK
4. Wolfson Centre for Applied Health Research, Bradford, UK
5. NIHR Applied Research Collaboration South-West Peninsula, Exeter, UK
6. Population Health Sciences Institute, Newcastle University, Newcastle upon Tyne, UK

**Supplementary Table 1 PRISMA-P (Preferred Reporting Items for Systematic review and Meta-Analysis Protocols) 2015 checklist**

| Section and topic | Item No | Checklist item | Reported on page # |
| --- | --- | --- | --- |
| ADMINISTRATIVE INFORMATION | | |  |
| Title: |  |  |  |
| Identification | 1a | Identify the report as a protocol of a systematic review | 1 |
| Update | 1b | If the protocol is for an update of a previous systematic review, identify as such | N/A |
| Registration | 2 | If registered, provide the name of the registry (such as PROSPERO) and registration number | N/A |
| Authors: |  |  |  |
| Contact | 3a | Provide name, institutional affiliation, e-mail address of all protocol authors; provide physical mailing address of corresponding author | 1-2 |
| Contributions | 3b | Describe contributions of protocol authors and identify the guarantor of the review | 22 |
| Amendments | 4 | If the protocol represents an amendment of a previously completed or published protocol, identify as such and list changes; otherwise, state plan for documenting important protocol amendments | N/A |
| Support: |  |  |  |
| Sources | 5a | Indicate sources of financial or other support for the review | 22 |
| Sponsor | 5b | Provide name for the review funder and/or sponsor | 22 |
| Role of sponsor or funder | 5c | Describe roles of funder(s), sponsor(s), and/or institution(s), if any, in developing the protocol | 22 |
| INTRODUCTION | | |  |
| Rationale | 6 | Describe the rationale for the review in the context of what is already known | 8-9 |
| Objectives | 7 | Provide an explicit statement of the question(s) the review will address with reference to participants, interventions, comparators, and outcomes (PICO) | 9-10 |
| METHODS | | |  |
| Eligibility criteria | 8 | Specify the study characteristics (such as PICO, study design, setting, time frame) and report characteristics (such as years considered, language, publication status) to be used as criteria for eligibility for the review | 10-13 |
| Information sources | 9 | Describe all intended information sources (such as electronic databases, contact with study authors, trial registers or other grey literature sources) with planned dates of coverage | 13-14 |
| Search strategy | 10 | Present draft of search strategy to be used for at least one electronic database, including planned limits, such that it could be repeated | 14, Supplementary |
| Study records: |  |  |  |
| Data management | 11a | Describe the mechanism(s) that will be used to manage records and data throughout the review | 15-16 |
| Selection process | 11b | State the process that will be used for selecting studies (such as two independent reviewers) through each phase of the review (that is, screening, eligibility and inclusion in meta-analysis) | 16-17 |
| Data collection process | 11c | Describe planned method of extracting data from reports (such as piloting forms, done independently, in duplicate), any processes for obtaining and confirming data from investigators | 17-19 |
| Data items | 12 | List and define all variables for which data will be sought (such as PICO items, funding sources), any pre-planned data assumptions and simplifications | 17-19 |
| Outcomes and prioritization | 13 | List and define all outcomes for which data will be sought, including prioritization of main and additional outcomes, with rationale | N/A |
| Risk of bias in individual studies | 14 | Describe anticipated methods for assessing risk of bias of individual studies, including whether this will be done at the outcome or study level, or both; state how this information will be used in data synthesis | N/A |
| Data synthesis | 15a | Describe criteria under which study data will be quantitatively synthesised | N/A |
|  | 15b | If data are appropriate for quantitative synthesis, describe planned summary measures, methods of handling data and methods of combining data from studies, including any planned exploration of consistency (such as I^2^, Kendall’s τ) | N/A |
|  | 15c | Describe any proposed additional analyses (such as sensitivity or subgroup analyses, meta-regression) | N/A |
|  | 15d | If quantitative synthesis is not appropriate, describe the type of summary planned | 19-20 |
| Meta-bias(es) | 16 | Specify any planned assessment of meta-bias(es) (such as publication bias across studies, selective reporting within studies) | N/A |
| Confidence in cumulative evidence | 17 | Describe how the strength of the body of evidence will be assessed (such as GRADE) | N/A |

Supplementary Table 2: Alphabetical list of websites included in grey literature searches

1. Condition specific websites

| Number | Organisation | Website used in the search |
| --- | --- | --- |
| 1 | All-Party Parliamentary Group on Dementia (UK) | alzheimers.org.uk/about-us/policy-and-influencing/all-party-parliamentary-group-dementia |
| 2 | All-Party Parliamentary Group on MND (UK) | mndassociation.org/get-involved/campaigning/all-party-parliamentary-group-appg-on-mnd |
| 3 | All-Party Parliamentary Group on Parkinson's (UK) | parkinsons.org.uk/get-involved/parkinsons-uk-parliament |
| 4 | All-Party Parliamentary Group on Rare, Genetic and Undiagnosed Conditions (UK) | geneticalliance.org.uk/appg/ |
| 5 | ALS Society of Canada | als.ca |
| 6 | ALS Therapy Development Institute (USA) | als.net |
| 7 | Alzheimer Europe | alzheimer-europe.org |
| 8 | Alzheimer Scotland | alzscot.org |
| 9 | Alzheimer Society of Canada | alzheimer.ca |
| 10 | Alzheimer's Association (USA) | alz.org |
| 11 | Alzheimer's Disease International | alzint.org |
| 12 | Alzheimer's Research UK | alzheimersresearchuk.org |
| 13 | Alzheimer's Society (UK) | alzheimers.org.uk |
| 14 | Alzheimer's Society of Ireland | alzheimer.ie |
| 15 | American Parkinson Disease Association | apdaparkinson.org |
| 16 | Davis Phinney Foundation for Parkinson's (USA) | davisphinneyfoundation.org |
| 17 | Dementia Australia | dementia.org.au |
| 18 | Dementia Society of America | dementiasociety.org |
| 19 | Dementia UK | dementiauk.org |
| 20 | European Huntington Association | eurohuntington.org |
| 21 | European Huntington's Disease Network | ehdn.org |
| 22 | Fight MND (Australia) | fightmnd.org.au |
| 23 | Huntington Society of Canada | huntingtonsociety.ca |
| 24 | Huntington's Disease Association (UK) | hda.org.uk |
| 25 | Huntington's Disease Society of America | hdsa.org |
| 26 | Huntington's Disease Tasmania | huntingtonstasmania.org.au |
| 27 | Huntington's Disease Youth Organisation (USA & UK) | hdyo.org |
| 28 | Huntington's Victoria (Australia) | huntingtonsvic.org.au |
| 29 | Huntington's Western Australia | huntingtonswa.org.au |
| 30 | International Alliance of ALS/MND Associations | als-mnd.org |
| 31 | International Huntington's Disease Association | huntington-disease.org |
| 32 | International Parkinson and Movement Disorder Society | movementdisorders.org |
| 33 | Irish Motor Neurone Disease Association | imnda.ie |
| 34 | Les Turner ALS Foundation (USA) | lesturnerals.org |
| 35 | Lewy Body Dementia Association (USA) | lbda.org |
| 36 | Lewy Body Dementia Canada | lewybodydementia.ca |
| 37 | Lewy Body Ireland | lewybodyireland.org |
| 38 | Lewy Body Society (UK) | lewybody.org |
| 39 | MND Scotland | mndscotland.org.uk |
| 40 | Motor Neurone Disease Association (UK) | mndassociation.org |
| 41 | National Institute of Neurological Disorders and Stroke (USA) | ninds.nih.gov |
| 42 | New South Wales HD Association (Australia) | huntingtonsnsw.org.au |
| 43 | Parkinson Canada | parkinson.ca |
| 44 | Parkinson's Australia | parkinsons.org.au |
| 45 | Parkinson's Europe | parkinsonseurope.org |
| 46 | Parkinson's Foundation (USA) | parkinson.org |
| 47 | Parkinson's Ireland | parkinsons.ie |
| 48 | Parkinson's UK | parkinsons.org.uk |
| 49 | Queensland HD Association (Australia) | huntingtonsqld.org.au |
| 50 | Rare Dementia Support (UK) | raredementiasupport.org |
| 51 | Scottish Huntington's Association | hdscotland.org |
| 52 | Shake It Up Foundation (Australia) | shakeitup.org.au |
| 53 | South Australia and Northern Territory HD Association | huntingtonssant.org.au |
| 54 | The ALS Association (USA) | als.org |
| 55 | World Dementia Council | worlddementiacouncil.org |

1. Non-condition-specific websites

| Number | Organisation | website |
| --- | --- | --- |
| 56 | AARP (USA) | aarp.org |
| 57 | Age UK | ageuk.org.uk |
| 58 | Ageing well Without Children (UK) | awwoc.org |
| 59 | Association of British Neurologists | theabn.org |
| 60 | British Psychological Society | bps.org.uk |
| 61 | British Society of Gerontology | britishgerontology.org |
| 62 | Centers for Disease Control and Prevention (USA) | cdc.gov |
| 63 | Centre for Ageing Better (UK) | ageing-better.org.uk |
| 64 | Health Policy Partnership (UK) | healthpolicypartnership.com |
| 65 | International Federation on Ageing | ifa.ngo |
| 66 | Joseph Rowntree Foundation (UK) | jrf.org.uk |
| 67 | Meaningful Ageing Australia | meaningfulageing.org.au |
| 68 | Neurological Alliance (UK) | neural.org.uk |
| 69 | NHS England | england.nhs.uk |
| 70 | Royal College of General Practitioners (UK) | rcgp.org.uk |
| 71 | Royal College of Occupational Therapists (UK) | rcot.co.uk |
| 72 | Royal College of Psychiatrists (UK) | rcpsych.ac.uk |
| 73 | The Health Foundation (UK) | health.org.uk |
| 74 | The King's Fund (UK) | kingsfund.org.uk |
| 75 | UK Government | service.gov.uk |
| 76 | World Health Organization | who.int |

Supplementary Table 3: Specific search strings to be used in applicable search databases

| Database | Search string |
| --- | --- |
| PubMed | (((((((((((((((((((dement*[Title/Abstract]) OR (Alzheimer*[Title/Abstract])) OR (Parkinson*[Title/Abstract])) OR (Lewy[Title/Abstract])) OR (Fronto*[Title/Abstract])) OR (Parkinsonism[Title/Abstract])) OR (Huntington*[Title/Abstract])) OR (Chorea[Title/Abstract])) OR (amyotrophic lateral sclerosis[Title/Abstract])) OR (ALS[Title/Abstract])) OR (motor neuron* disease[Title/Abstract])) OR (MND[Title/Abstract])) OR (progressive muscular atrophy[Title/Abstract])) OR (Gehrig[Title/Abstract]) OR (neurodegen*[Title/Abstract]) OR (neurolog*[Title/Abstract]) OR (cognitive impairment[Title/Abstract]))))))) AND (((((((((Living alone[Title/Abstract]) OR Live* alone[Title/Abstract]) OR (Single*living[Title/Abstract])) OR (One-person household[Title/Abstract])) OR (Singlehood[Title/Abstract])) OR (Single people[Title/Abstract])) OR (Single person[Title/Abstract])) OR (Single men[Title/Abstract])) OR (Single women[Title/Abstract])) OR (Solo[Title/Abstract]) |
| Web of Science | (TI=(dement* OR Alzheimer* OR Parkinson* OR Lewy OR Fronto* OR Parkinsonism OR Huntington* OR Chorea OR amyotrophic lateral sclerosis OR ALS OR motor neuron* disease OR MND OR progressive muscular atrophy OR Gehrig OR neurodegen* OR neurolog* OR cognitive impairment)) AND (TI=(Living alone OR Live* alone OR Single-living OR One-person household OR Singlehood OR Single people OR Single person OR Single men OR Single women OR solo)) OR (AB=(dement* OR Alzheimer* OR Parkinson* OR Lewy OR Fronto* OR Parkinsonism OR Huntington* OR Chorea OR amyotrophic lateral sclerosis OR ALS OR motor neuron* disease OR MND OR progressive muscular atrophy OR Gehrig OR neurodegen* OR neurolog* OR cognitive impairment)) AND (AB=(Living alone OR Live* alone OR Single-living OR One-person household OR Singlehood OR Single people OR Single person OR Single men OR Single women OR solo)) |
| EBSCOhost (CINAHL and Ageline) | TI ( dementia OR Alzheimer* OR Parkinson* OR Lewy OR Fronto OR Parkinsonism OR Huntington OR Chorea OR amyotrophic lateral sclerosis OR ALS OR motor neuron disease OR MND OR progressive muscular atrophy OR Gehrig OR neurodegen* OR neurol* ) AND TI ( Living alone OR Single-living OR One-person household OR Singlehood OR Single people OR Single person OR Single men OR Single women OR solo ) OR AB ( dementia OR Alzheimer* OR Parkinson* OR Lewy OR Fronto OR Parkinsonism OR Huntington OR Chorea OR amyotrophic lateral sclerosis OR ALS OR motor neuron disease OR MND OR progressive muscular atrophy OR Gehrig OR neurodegen* OR neurol* ) AND AB ( Living alone OR Single-living OR One-person household OR Singlehood OR Single people OR Single person OR Single men OR Single women OR solo ) |
| Ovid (EMBASE, PsycInfo, and Social Policy and Practice) | ((dement* or Alzheimer* or Parkinson* or Lewy or Fronto* or Parkinsonism or Huntington* or Chorea or amyotrophic lateral sclerosis or ALS or motor neuron* disease or MND or progressive muscular atrophy or Gehrig or neurodegen* or neurolog* or cognitive impairment) and (Living alone or Live* alone or Single-living or One-person household or Singlehood or Single people or Single person or Single men or Single women or solo)).ti,ab. |
