# Supplementary material for: Examining the challenges of people living alone with neurodegenerative conditions: a scoping review protocol
Source: BMJ Open. 2025 Jun 10;15(6):e090146. doi: 10.1136/bmjopen-2024-090146 (PMC12161305; doi:10.1136/bmjopen-2024-090146)
Supplement: Supplementary file 2 [file bmjopen-2024-090146.reviewer_comments.pdf]

# PEER REVIEW HISTORY

BMJ Open publishes all reviews undertaken for accepted manuscripts. Reviewers are asked to complete a checklist review form and are provided with free text boxes to elaborate on their assessment. These free text comments are reproduced below.

## ARTICLE DETAILS

### Title (Provisional)

Examining the challenges of people living alone with neurodegenerative conditions: a scoping review protocol.

### Authors

Martyr, Anthony; Caulfield, Maria; Charlwood, Catherine; Gamble, Laura D; Prina, Matthew; Oyebode, Jan R; Hulme, Claire; Clare, Linda

---

## VERSION 1 - REVIEW

---

| | |
|---|---|
| **Reviewer** | **1** |
| **Name** | **Bacsu, Juanita-Dawne** |
| **Affiliation** | **Thompson Rivers University, School of Nursing** |
| **Date** | **03-Feb-2025** |
| **COI** | **None** |

---

February 3, 2025

Dear BMJ Open Editorial Committee,

Thank you for the opportunity to review the article entitled, “People living alone with neurodegenerative conditions: a scoping review protocol.” This article focuses on providing a scoping review protocol to outline the steps that will be taken in the full scoping review on people living alone with neurocognitive conditions. I have included my feedback for the authors, and I hope they find my comments useful.

1. The title of the article could be strengthened with some revision to help identify the aim of the study. For example, the title could be revised to something like, "Examining the challenges of people living alone with neurocognitive conditions: A scoping review protocol."

2. In the abstract, it would be helpful to identify the purpose of this scoping review protocol in addition to discussing the aim of the scoping review.

3. In the abstract’s methods section, consider tightening and refining the methods. The feasibility of the study is a bit concerning since no timeframe will be included for the search and the authors also plan to search peer-reviewed and grey literature.

4. In the abstract's ethics and dissemination section, please clarify why ethics is not required especially if people with lived experience are participating in consultations to help guide the study.

5. It would be helpful for the authors to include a few more words surrounding the greater risk of exploitation (page 7, line 12) to provide clarity.

6. Considering rephrasing “will” with “may” in the following statement:

“Synthesising available evidence will result in recommendations that can inform the development of policies and services tailored to meet the diverse needs of people with neurodegenerative conditions who live alone.”

7. Identification of the primary research question would be helpful in the section of the research objective. The research objective section would have improved flow if it was rewritten without the usage of bullet points.

8. In the methods, it would be helpful to include discussion about why no timeframe is being selected for the search.

9. Page 9, line 31-36 bullets would be useful at the end of the article to reflect on the importance of the review at the end of the scoping review protocol.

10. Pages 10-11, would be strengthened by restructuring the bullet points into a manuscript style article without the usage of bullet points to provide better flow throughout the protocol.

11. Page 13, lines 17-20 “were” should be changed to “will” unless the review has already been conducted.

12. It would be helpful to discuss whether an expert librarian was consulted to aid in the review.

13. Search terms should be included in a table format rather than bullet points. Also, the search strategy would benefit from more refinement to ensure the proposed search is feasible. For example, the feasibility of scoping review seems rather extensive given that no timeframe is being used. Consultation with an expert librarian would aid in the refinement of the review’s search strategy.

14. Page 15, line 23, it would be helpful for the authors to discuss why they are choosing excel to manage the scoping review data rather than software created for reviews such as Covidence.

15. Page 15, line 42, it would be helpful for the authors to discuss if they will be providing a figure to show the filtering of the scoping review results

16. Page 16, line 15-26 should be included by the section on inclusion criteria earlier in the manuscript.

17. Page 17-18, lines 17 -8, should be rewritten without the usage of bullet points for enhanced article flow.

18. Page 19, lines 31, section on stakeholder consultation would be strengthened by included discussion about how people with lived experience will be selected as a consultant on the study and how they will participate.

19. Page 19, ethics and dissemination section needs to include more information surrounding why ethics is not required, especially if people with lived experience are participating in consultations. Additionally, more information on the dissemination strategy would be helpful.

20. Overall, the article would be strengthened from removing the bullet points to improve the flow and consistency throughout the paper. Additionally, consider adding sub-headings to enhance clarity and help guide the reader through the different steps of the scoping review protocol. The structure of the article feels a bit unclear and disorganized at times, particularly with the inclusion criteria being discussed in the data synthesis section. Refining the order and adding sub-headings would strengthen the clarity of the paper.

| | |
|---|---|
| **Reviewer** | **2** |
| **Name** | **Persson, Ann-Christine** |
| **Affiliation** | **Karolinska Institutet** |
| **Date** | **28-Apr-2025** |
| **COI** | **None** |

Dear Editor,

Thank you for inviting me to this review of the manuscript entitled "People living alone with neurodegenerative conditions: a scoping review protocol."

Comments to authors:

The aim of this scoping review protocol is to systematically assess both research and grey literature concerning people with neurodegenerative conditions who live alone in the community. Increased knowledge on the target group's challenges and needs can be used to better inform health and social care services and enable more tailored and appropriate service delivery.

This is an important and urgent subject. There is a need for increased knowledge about the challenges and needs of a vulnerable group for both researchers, professionals and decision-makers. The study protocol is comprehensive, well-designed and detailed. However, the

scope is wide and somewhat difficult to grasp, why the protocol could benefit from using concepts more stringent in background, rationale and aim. For example, independence is used only in the abstract and not in the background.

Also, there is no information on when the review will be undertaken?

Minor comments:

Strengths and limitations of this study

Page 4, lines 36-38: Revise sentence structure. Do you mean: "A comprehensive search in seven databases will maximise identification of all possible records that meet the inclusion criteria of the review"?

Background

Page 5, lines 22-24: Which factors are supposed to be more salient for older adults?

Stakeholder consultation

Page 19, lines 31-33: How were the stakeholders involved and how did they influence the planning of the study?

---

## VERSION 1 - AUTHOR RESPONSE

Reviewer: 1
Dr. Juanita-Dawne Bacsu, Thompson Rivers University
Comments to the Author:
February 3, 2025
Dear BMJ Open Editorial Committee,
Thank you for the opportunity to review the article entitled, "People living alone with neurodegenerative conditions: a scoping review protocol." This article focuses on providing a scoping review protocol to outline the steps that will be taken in the full scoping review on people living alone with neurocognitive conditions. I have included my feedback for the authors, and I hope they find my comments useful.

1. The title of the article could be strengthened with some revision to help identify the aim of the study. For example, the title could be revised to something like, "Examining the challenges of people living alone with neurocognitive conditions: A scoping review protocol."

*Thanks for this suggestion, the title has been amended as suggested.*

2. In the abstract, it would be helpful to identify the purpose of this scoping review protocol in addition to discussing the aim of the scoping review.

*We have added a sentence about the purpose of the protocol to make the methodology and processes that we used in our subsequent scoping review transparent and reproducible.*

3. In the abstract's methods section, consider tightening and refining the methods. The

feasibility of the study is a bit concerning since no timeframe will be included for the search and the authors also plan to search peer-reviewed and grey literature.

*Thanks for allowing us the opportunity to clarify this point. As there was no previous review on the topic, as far as we know, the search was unrestricted regarding timeframe. Piloting of the search strategy provided an estimate of the number of titles that were likely needed to be title and abstract screened. The piloting and prior experience in a previous review that used a similar strategy meant that the search strategy was deemed feasible. The size of the research team also meant that different people could devote time to different elements of the search and screening strategy with different groups working on searching and screening the published literature and the grey literature. The methods section has not been amended as this was the process that we followed in the subsequent review.*

4. In the abstract's ethics and dissemination section, please clarify why ethics is not required especially if people with lived experience are participating in consultations to help guide the study.

*As stakeholders with lived experience are partners in the review and the overarching Policy Research Unit programme of work rather than participants in research data collection ethical approval is not needed. This was informed by consultation with our ethical committee and our experiences from previous research programmes.*

5. It would be helpful for the authors to include a few more words surrounding the greater risk of exploitation (page 7, line 12) to provide clarity.

*We have added some words to clarify that when exploitation occurs it is often financial exploitation.*

6. Considering rephrasing "will" with "may" in the following statement:
"Synthesising available evidence will result in recommendations that can inform the development of policies and services tailored to meet the diverse needs of people with neurodegenerative conditions who live alone."

*We changed 'will' to "is expected to"*

7. Identification of the primary research question would be helpful in the section of the research objective. The research objective section would have improved flow if it was rewritten without the usage of bullet points.

*We have now signposted the primary research question of the eventual scoping review.*

8. In the methods, it would be helpful to include discussion about why no timeframe is being selected for the search.

*As no previous review of living alone with the included conditions exists, as far as we are aware, it was felt that to be as inclusive as possible there should not be any date restrictions applied to the searches. A statement about this has been included where the search date was discussed.*

9. Page 9, line 31-36 bullets would be useful at the end of the article to reflect on the importance of the review at the end of the scoping review protocol.

*We have removed the bullet points here, as suggested.*

10. Pages 10-11, would be strengthened by restructuring the bullet points into a manuscript style article without the usage of bullet points to provide better flow throughout the protocol.

*Bullet points were retained here so that the eligibility criteria can be more easily found by an interested reader, as we feel that the inclusion and exclusion criteria are important and should be bulleted so that each point stands out.*

11. Page 13, lines 17-20 "were" should be changed to "will" unless the review has already been conducted.

*This specific line discusses the search terms used in the scoping review, we used the word "were" here as the search terms are included in the reviewed protocol so had already been collated for inclusion in the protocol.*

12. It would be helpful to discuss whether an expert librarian was consulted to aid in the review.

*The review team comprise very experienced reviewers and a search strategy was developed for all DeNPRU Exeter reviews; this was based on previous discussions with expert librarians. Consequently, the search strategy described in the protocol was not directly discussed with an expert librarian, but the strategy was based on previous discussions and reviews that did benefit from the input of a librarian. In addition, the search strategy included in the scoping review was based on one previously used by the authors: Martyr et al, 2018 https://doi.org/10.1017/S0033291718000405*

13. Search terms should be included in a table format rather than bullet points. Also, the search strategy would benefit from more refinement to ensure the proposed search is feasible. For example, the feasibility of scoping review seems rather extensive given that no timeframe is being used. Consultation with an expert librarian would aid in the refinement of the review's search strategy.

*The search terms have been included in Table 1. As mentioned above, the search strategy was deemed feasible based on previous experience of conducting similar reviews and prior discussions with librarians, as well as by piloting the search terms to estimate likely workload. The number of returns found in the search was manageable, and the review was completed on time.*

14. Page 15, line 23, it would be helpful for the authors to discuss why they are choosing excel to manage the scoping review data rather than software created for reviews such as Covidence.

*We mention using Excel here as this specific sentence was discussing how we were going to manage and record information about the grey literature searches only. Using Covidence for this process would have meant manually importing the citation for each downloaded pdf,*

*which would have added a significant amount of time to the grey literature search procedure. We have added "grey literature" to this sentence to make it clear that Excel was being used to record the grey literature searches only.*

15. Page 15, line 42, it would be helpful for the authors to discuss if they will be providing a figure to show the filtering of the scoping review results

*We have added a statement that a PRISMA flowchart will be included that maps the screening and study selection stages.*

16. Page 16, line 15-26 should be included by the section on inclusion criteria earlier in the manuscript.

*Thanks for this point. We have removed parts of this text as it duplicated what was included in the eligibility criteria section.*

17. Page 17-18, lines 17 -8, should be rewritten without the usage of bullet points for enhanced article flow.

*We have removed the bullet points here, as suggested.*

18. Page 19, lines 31, section on stakeholder consultation would be strengthened by included discussion about how people with lived experience will be selected as a consultant on the study and how they will participate.

*We have added sentences clarifying how people with lived experience will act as consultees through participation in online workshops to discuss experiences of living alone and/or caring at a distance.*

19. Page 19, ethics and dissemination section needs to include more information surrounding why ethics is not required, especially if people with lived experience are participating in consultations. Additionally, more information on the dissemination strategy would be helpful.

*We have added sentences clarifying that no ethics were required because people with lived experience were not participating in research, but had elected to join consultation workshops whereby they are asked to share their own experiences and opinions. We have added a sentence clarifying the dissemination strategy.*

20. Overall, the article would be strengthened from removing the bullet points to improve the flow and consistency throughout the paper. Additionally, consider adding sub-headings to enhance clarity and help guide the reader through the different steps of the scoping review protocol. The structure of the article feels a bit unclear and disorganized at times, particularly with the inclusion criteria being discussed in the data synthesis section. Refining the order and adding sub-headings would strengthen the clarity of the paper.

*We removed most of the bullet points as suggested by the reviewer, we retained the bullets for the eligibility criteria section as we feel these are easier to follow when formatted this way. For the section headings we followed and adhered to the structure, order, and section headings that are outlined in the PRISMA-P checklist, with the addition at the end of the protocol of the stakeholder consultation and dissemination and ethics sections which are not*

*included in the PRISMA guidelines for protocols but were included as this information and procedures are important. Eligibility was only included in the eligibility criteria section and not in the data synthesis section.*

Reviewer: 2
Dr. Ann-Christine Persson, Karolinska Institutet
Comments to the Author:
Dear Editor,
Thank you for inviting me to this review of the manuscript entitled "People living alone with neurodegenerative conditions: a scoping review protocol."
Comments to authors:
The aim of this scoping review protocol is to systematically assess both research and grey literature concerning people with neurodegenerative conditions who live alone in the community. Increased knowledge on the target group's challenges and needs can be used to better inform health and social care services and enable more tailored and appropriate service delivery.
This is an important and urgent subject. There is a need for increased knowledge about the challenges and needs of a vulnerable group for both researchers, professionals and decision-makers. The study protocol is comprehensive, well-designed and detailed. However, the scope is wide and somewhat difficult to grasp, why the protocol could benefit from using concepts more stringent in background, rationale and aim. For example, independence is used only in the abstract and not in the background.

*Thanks for this point, we have made it clearer in the introduction that living independently and independence are important elements of living alone.*

Also, there is no information on when the review will be undertaken?

*The date by which the work was expected to be completed has been added to the end of the protocol.*

Minor comments:
Strengths and limitations of this study
Page 4, lines 36-38: Revise sentence structure. Do you mean: "A comprehensive search in seven databases will maximise identification of all possible records that meet the inclusion criteria of the review"?

*Thanks for this point, we have amended this sentence as suggested.*

Background
Page 5, lines 22-24: Which factors are supposed to be more salient for older adults?

*The primary factor for older people living alone is the death of a spouse or partner.*

Stakeholder consultation
Page 19, lines 31-33: How were the stakeholders involved and how did they influence the planning of the study?

*We have added detail around this point. On the review team we had two stakeholders, one person who was a carer at a distance for a parent who lived alone with dementia, and*

*another person who lives alone with Parkinson's disease. They have attended each project meeting as these were all online, and they contributed to discussions around the review topic and influenced the direction of the project.*
